# *Lactobacillus*-Based Probiotics Reduce the Adverse Effects of Stress in Rodents: A Meta-analysis

**DOI:** 10.3389/fnbeh.2021.642757

**Published:** 2021-06-16

**Authors:** Claire Mindus, Jennifer Ellis, Nienke van Staaveren, Alexandra Harlander-Matauschek

**Affiliations:** Department of Animal Biosciences, University of Guelph, Guelph, ON, Canada

**Keywords:** meta-analysis, probiotic, *Lactobacillus*, stress, psychological disorder, gut-brain axis

## Abstract

*Lactobacillus* species play a critical role in the bidirectional communication between the gut and the brain. Consequently, they have the potential to aid in the treatment of psychological disorders. The impact of *Lactobacillus* supplementation on the stress responses triggering psychological disorders has not been systematically reviewed. Therefore, the aim of this meta-analysis is to summarize the body of research assessing the effects of *Lactobacillus*-based probiotics in rodents that underwent an experimental stress treatment or not. The duration of immobility in a Forced Swim Test (FST) was the outcome used to measure changes induced by various treatments. Four online databases were systematically searched for relevant studies published in English. Fourteen studies meeting the criteria were included in the meta-analysis. The effects of probiotic supplementation and stress treatment on the duration of immobility in the FST were analyzed using a generalized linear mixed model. Publication bias was evaluated by funnel plots. Our analysis shows that *Lactobacillus*-based probiotic supplements significantly reduce immobility in the FST (*P* < 0.001) in stressed rodents. However, probiotics did not affect the rodents that did not undergo the stress treatment (*P* = 0.168). These findings provide a better understanding of the potential of *Lactobacillus*-based probiotics for the management of stress-induced behavior.

## Introduction

*Lactobacillus* species have a long history of use by humans (Holzapfel, 2002) and are considered safe by the World Health Organization (WHO and FAO, 2006). For example, *Lactobacillus* species are best known for their lactic acid production used to produce cheese and other fermented foods (Briggiler-Marcó et al., 2007). Despite representing a minor proportion of the human gut microbiota (Nistal et al., 2016; Almonacid et al., 2017), increased or depleted *Lactobacillus* populations are associated with states of health and disease (Heeney et al., 2018; Zhang et al., 2018). More specifically, *Lactobacillus* species are reported to impart beneficial effects on the stress response and the immune system when used as a probiotic (Bravo et al., 2011; Palomar et al., 2014; Huang et al., 2016; Lew et al., 2019). Because of these health-promoting characteristics, they have become the focus of several gut microbiome studies in mammals (Zhang et al., 2018) which have paved the way for the potential use of *Lactobacillus*-based therapies to treat or prevent stress-induced psychological disorders (Huang et al., 2016; Lowry et al., 2016; Marin et al., 2017; Ng et al., 2018; Reis et al., 2018; Aizawa et al., 2019), such as depression and anxiety disorders that impact up to 8% of the world population (World Health Organization, 2017).

A complex and bidirectional communication network exists between the gut and the central nervous system, which includes the enteric nervous system (ENS), the immune system and the modulation of neuroactive compounds by the microbiota (Grenham et al., 2011; Holzer and Farzi, 2014). Under stress, the microbiota is proposed to influence the central nervous system via the immune system and ENS (Foster and McVey Neufeld, 2013; Huang et al., 2016; Foster et al., 2017; Wallace and Milev, 2017). Indeed, *Lactobacillus* species are known to generate neuroactive and neuroimmune substances such as acetylcholine (Marquardt and Spitznagel, 1959; Stanaszek et al., 1977), nitric oxide (Sobko et al., 2006), histamine (Özogul et al., 2012; Thomas et al., 2012), as well as the neurotransmitters serotonin, dopamine (Özogul et al., 2012; Liu et al., 2016) and gamma-aminobutyric acid (GABA) (Yokoyama et al., 2002; Cho et al., 2007; Bravo et al., 2011). Therefore, *Lactobacillus* species may have the potential to prevent or aid in treatment of psychological disorders. To this end, a number of animal trials and recent reviews have investigated the impact of probiotic consumption on behavior related to psychological disorders.

Standardized behavioral tests, such as the forced swim test (FST), the sucrose preference test, or the tail suspension test, are routinely used in rodent models to assess the antidepressant potential of various compounds. For example, Bravo et al. (2011) demonstrated that ingesting *Lactobacillus rhamnosus* JB-1 reduces stress-induced immobility duration in the FST in adult male mice. The use of these animal models are largely pragmatic and they are not designed to fully represent psychological disorders (Gururajan et al., 2019; Reardon, 2019). Regardless, they can provide valuable information to understand the exact mechanisms through which *Lactobacillus* bacteria modulate gut-brain communication, as the exact role of the microbiota in psychological disorders remains poorly defined. To the best of our knowledge, no previous publication has systematically reviewed the effects of *Lactobacillus*-based supplementation on the behavioral outcomes of stressed vs. non-stressed rodent populations as measured by standard tests. Therefore, we hypothesize that, under experimental stressful conditions, rodents receiving a *Lactobacillus*-based supplement will demonstrate a shorter immobility duration measured in the FST compared to counterparts that receive a control supplement. A meta-analysis of published literature recording behavioral outcomes using the FST following a stress regimen in *Lactobacillus*-based supplement vs. control rodents were used to validate this hypothesis.

## Materials and Methods

### Search Strategy

Protocols employed were based on the PRISMA guidelines. The goal of this analysis was to study the effect of *Lactobacillus* species supplementation and stress treatments on rodent behavior measured in standardized tests. ProQuest, ScienceDirect, Web of Science, and PubMed databases were systematically searched in January and February 2020. The search terms included: (*Lactobacillus* OR Lactobacilli OR Lactic acid bacteria) AND (stress OR stressful OR fear OR fearful OR psychological OR restraint OR social defeat NOT oxidative) AND (murine OR rodent OR rodents OR mouse OR mice OR rat OR rats) AND (mood disorder OR depression OR depressive OR anxiety OR stress behavior) in titles or abstracts without imposing time limitations. We also reviewed the references provided in each publication, as well as those provided in systematic review articles associated with mental health and stress-induced psychological or physical disorders.

Studies were deemed eligible if they met the following inclusion criteria:

- English language articles,
- Studies published in peer-reviewed journals with a randomized control group,
- Studies used mice or rats (not germ-free animals),
- Tested animals were directly supplemented with live *Lactobacillus* species (without any limitations on culture, strain, dose, or therapy regimen) as an experimental treatment (e.g., if the supplement was given to the mother of infant rodent, the study was excluded),
- A second experimental treatment included a stress directly administered to the individual or via a change in its environment. There were no limitations on category and numbers of stressors used, their frequency, duration and the order of the stressors compared to the *Lactobacillus* species supplementation,
- Following the supplementation and stress treatments, behavioral outcomes were measured in standardized tests commonly used in rodent models [i.e., the forced swim test (FST), the sucrose preference test, or tail suspension test]. These tests themselves can be considered as acute stressors for the animals (Commons et al., 2017). However, in this study, all animals underwent these standardized tests, regardless of whether they were in the experimental stressed group (“Stress”) or not (“Non-stress”) as described in criteria 5. As such, the standardized behavioral tests were considered separate from the experimental stress treatment

### Literature Search and Screening

**Figure 1:**
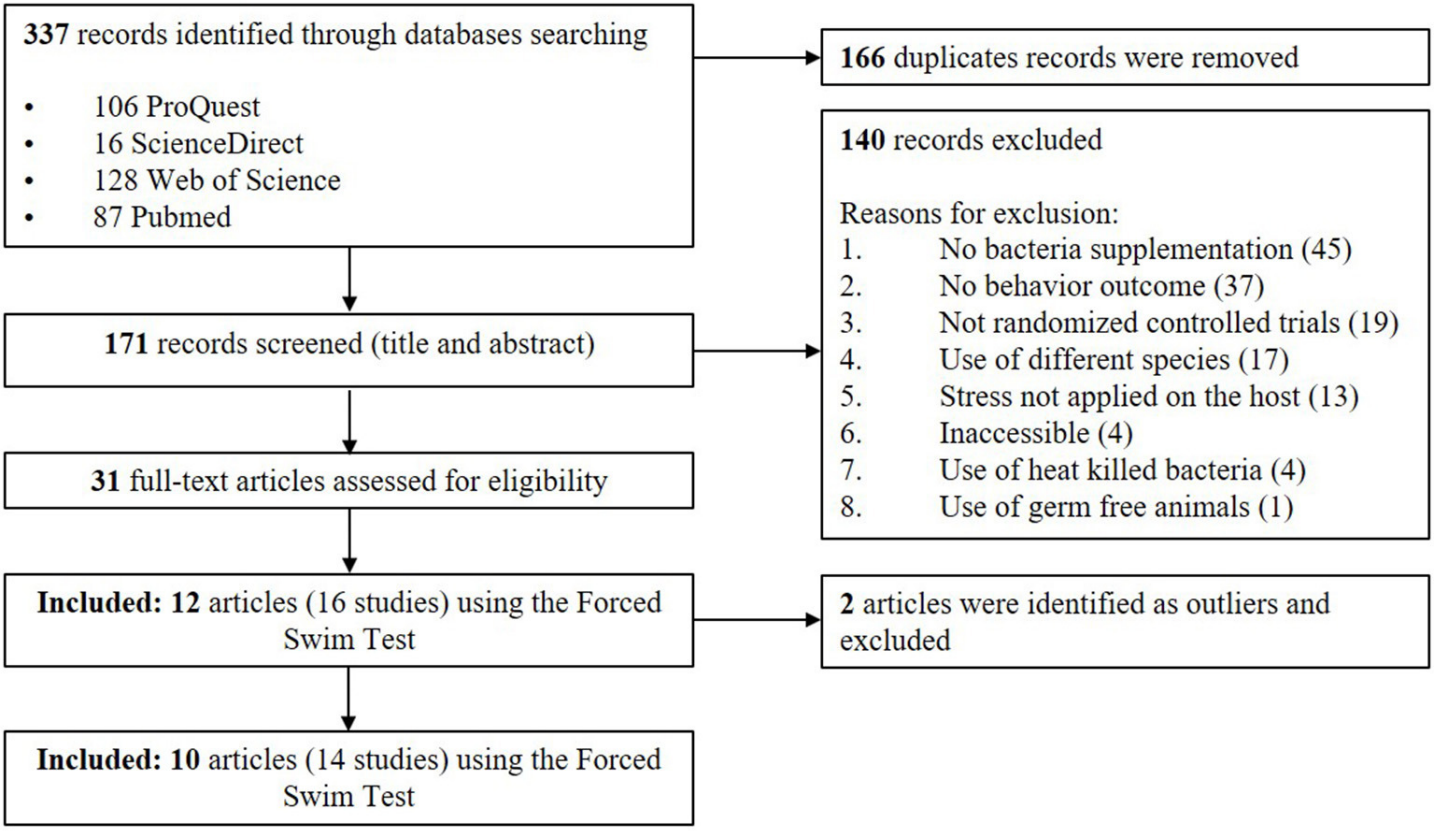
PRISMA flow diagram of studies included in meta-analysis—*Lactobacillus*-based probiotics reduce the adverse effects of stress in rodents.

An adapted PRISMA flow diagram was used to represent the process of article and study selection for our meta-analysis (Figure 1). We did not use a pre-registered protocol for the systematic review and meta-analysis. In our initial search, 337 English language records, were identified which, after screening, revealed 171 unique articles. A total of 31 articles fulfilled the selection criteria using various standardized behavioral tests but the largest number of articles used the FST. The FST involves placing an animal in an inescapable container filled with water. The animal initially displays an active coping strategy, including behaviors such as climbing or swimming. This is followed by a passive coping strategy where they stay immobile and floating (Porsolt et al., 1977). For the current meta-analysis, we ultimately chose to focus on the articles measuring the immobility duration in the FST. This test is the most widely used screening tool in rodent models for the purpose of identifying novel antidepressant compounds (Cryan and Holmes, 2005; Kara et al., 2018). Furthermore, this strategy would allow collection of a standardized outcome (immobility duration) needed to perform the meta-analysis (Kara et al., 2018). However, it should be acknowledged that the test is criticized for its cavalier use in depression research and its limitations are reviewed in the Discussion section (Molendijk and de Kloet, 2015; De Kloet and Molendijk, 2016; Reardon, 2019).

A total of 12 papers that fulfilled the selection criteria measuring the immobility duration in the FST after a *Lactobacillus*-based supplementation and a stress treatment were included in the quantitative meta-analysis. Stenman et al. (2020) and Jang et al. (2019) provided results from different 3 and 2 *Lactobacillus*-based probiotics supplementations, respectively; and Murray et al. (2019) used both males and females resulting in a total of 16 available studies for the meta-analysis. Characteristics of each study are shown in Table 1. Two articles were later excluded, as they contained individual points that were later (during statistical analysis) identified as outliers (Dhaliwal et al., 2018; Li et al., 2019). Thus, the final total number of studies retained in the final meta-analysis was 14 (Liu et al., 2016, 2019; Li et al., 2018; Morshedi et al., 2018; Jang et al., 2019; Liao et al., 2019; Murray et al., 2019; Sun et al., 2019; Wei et al., 2019; Stenman et al., 2020).

**Table 1:** Characteristics of studies collected from the literature review search for the meta-analysis on the effect of *Lactobacillus*-based supplementation and stress treatments in rodents in the Forced Swim Test (FST).

**References (Study #)**	**Subjects**	**Bacterial species, strain and dosage**	**Probiotic dose**	**Stress treatment**	**Treatments strategy**	**FST parameters**	**Other behavioral tests**
Morshedi et al. (2018) (1)	18 Males Wistar rats, 5–7 woa^d^	*L. plantarum* ATCC 8014 (10^7^ CFU/ml)	1 time/day for 49 days, via gastric gavage	STZ^a^ (35 mg/kg), once, via intraperitoneal injection	Reversal^f^	300 s long, in Plexiglas cylinder (40 cm depth, Ø^i^ 20 cm) filled with water at 24 ± 1 °C 24 h earlier 15min pretest	EPM^j^, FST
Murray et al. (2019) (2)	40 Males CD-1 mice, 3 woa	*L. lactis, L. cremoris, L. diacetylactis, L. acidophilus* (3.0 × 10^9^ CFU/g)	3 times/week for 21 days, via Kefir	LPS^b^ (1.5 mg/kg), once, via intraperitoneal injection	Protection^g^	300 s long, 4 L glass beaker filled with 3 L water at 24 ± 2°C No pretest	EPM, OFT^k^, Rotarod Test, FST
Murray et al. (2019) (3)	40 Females CD-1 mice, 3 woa	*L. lactis, L. cremoris, L. diacetylactis, L. acidophilus* (3.0 × 10^9^ CFU/g)	3 times/week for 21 days, via Kefir	LPS (1.5 mg/kg), once, via intraperitoneal injection	Protection	300 s long, in 4 L glass beaker filled with 3 L water at 24 ± 2°C No pretest	EPM, OFT^k^, Rotarod Test, FST
Li et al. (2018) (4)	32 Males C57BL/6 mice,6–8 woa	*L. helveticus* R0052, *L. plantarum* R1012, and *B. longum* R0175 (10 × 10^9^ CFU/mL)	1 time/day for 28 days, via oral gavage	Chronic Mild Stress, for 28 days, at variable frequencies	Protection	240 s long, in glass cylinder (23 cm depth, Ø 12 cm) filled with water at 24 ± 1°C No pretest	SPT^l^, EPM, FST
Stenman et al. (2020) (5)	47 Males Swiss mice, 5 woa	*L. paracasei* Lpc-37, *L. salivarius* Ls-33 (1 × 10^9^ CFU per day)	1 time/day for 35 days, via oral gavage	Chronic, for 21 days, 1 time/day	Prevention^h^	300 s long, Water at 22 ± 1°C in transparent cylinder (24 cm depth, Ø 12 cm) filled with 12 cm water at 22 ± 1°C No pretest	EPM, OFT, NOR^m^, FST
Stenman et al. (2020) (6)	58 Males Swiss mice, 5 woa	*L. plantarum* LP12418, *L. plantarum* LP12151, *L. plantarum* LP12407 (1 × 10^9^ CFU per day)	1 time/day for 35 days, via oral gavage	Chronic, for 21 days, 1 time/day	Prevention	300 s long, Water at 22 ± 1°C in transparent cylinder (24 cm depth, Ø 12 cm) filled with 12 cm water at 22 ± 1°C No pretest	EPM, OFT, NOR^m^, FST
Stenman et al. (2020) (7)	60 Male Swiss mice, 5 woa	*L. acidophilus* LA11873, *L. rhamnosus* LX11881 (DGCC11881), *L. helveticus* LH0138 (1 × 10^9^ CFU per day)	1 time/day for 35 days, via oral gavage	Chronic, for 21 days, 1 time/day	Prevention	300 s long, in transparent cylinder (24 cm depth, Ø 12 cm) filled with 12 cm water at 22 ± 1°C No pretest	EPM, OFT, NOR^m^, FST
Jang et al. (2019) (8)	28 Male C57BL/6 mice, 5 woa	*L. reuteri* NK33 (1 × 10^9^ CFU per day)	1 time/day for 5 days, via oral gavage	Immobilization, for 2 days, 1 time/day	Reversal	300 s long, in transparent plastic jar (40 cm depth) filled with 25 cm water at 25°C No pretest	EPM, LDT^n^, TST^o^, FST
Jang et al. (2019) (9)	28 Males C57BL/6 mice, 5 woa	*L. reuteri* NK33 and *B. adolescentis* NK98 (1 × 10^9^ CFU per day)	1 time/day for 5 days, via oral gavage	Immobilization, once	Prevention	300 s long, in transparent plastic jar (40 cm depth) filled with 25 cm water at 25°C No pretest	EPM, LDT^n^, TST^o^, FST
Liu et al. (2019) (10)	18 Males Wistar rats, 7–8 woa	*L. fermentum* PS150 (1 × 10^9^ CFU per day)	1 time/day for 28 days, via oral gavage	Chronic Mild Stress, for 28 days, 3 times/day	Protection	300 s long, Water at 23 ± 1°C in transparent glass container (50 cm depth and Ø 20 cm) 24 h earlier 15 min pretest	FST, OFT, EPM, MWMT^p^, NOR
Liu et al. (2016) (11)	32 Males C57BL/6J mice, 2 doa^e^	*L. plantarum* PS128 (1 × 10^9^ CFU per day)	1 time/day for 28 days, via oral gavage	Maternal separation, for 12 days, 1 time/day	Reversal	300 s long, in transparent acrylic cylinder (30 cm depth and Ø 20 cm) filled with 15 cm water at 24 ± 1°C No pretest	SPT, OFT, EPM, FST
Liao et al. (2019) (12)	20 Males C57BL/6J mice, 2 doa	*L. paracasei* PS23 (1 × 10^9^ CFU per day)	1 time/day for 28 days, via oral gavage	Maternal separation, for 12 days, 1 time/day	Reversal	360 s long, in transparent acrylic cylinder (30 cm depth and Ø 10 cm) filled with 15 cm water at 24 ± 1°C 24 h earlier 6 min pretest	OFT, EPM, FST
Wei et al. (2019) (13)	24 Males C57BL/6J mice, 6–8 woa	*L. paracasei* PS23 (5 × 10^8^ cells/ml)	1 time/day for 40 days, via oral gavage	Cort (40 mg/kg), for 20 days, 1 time/day, via subcutaneous injection	Prevention	360 s long, in acrylic cylinder (25 cm depth and Ø 9 cm) filled with 15 cm water at 24–25°C 48 h earlier 6.5 min pretest	OFT, FST, SPT
Sun et al. (2019) (14)	24 Males Kunming mice, adult	*L. kefiranofaciens* ZW3 (1 × 10^9^ CFU per day)	1 time/day for 7 days, via oral gavage	Chronic Unpredictable Mild Stress, for 42 days, 1 time/day	Reversal	240 s long, in transparent beaker (Ø 16 cm) filled with 10 cm water at 25°C 24 h earlier 15 min pretest	SPT, OFT, FST
Li et al. (2019)^c^ (15)	30 Males Wistar mice, adult	*L. rhamnosus* (1 × 10^9^ CFU/100 g weight)	1 time/day for 28 days, via oral gavage	Chronic Unpredictable Mild Stress, for 28 days, 1 time/day	Protection	300 s long in Perspex cylinder filled with 30 cm water at 25°C 24 h earlier 15 min pretest	FST, SPT
Dhaliwal et al. (2018)^c^ (16)	48 Males Swiss albino LACA mice, adult	*L. plantarum* MTCC 9510 (2 × 10^10^ CFU)	1 time/day for 28 days, via oral gavage	Chronic Unpredictable Mild Stress, for 28 days, 1 time/week	Protection	360 s long in rectangular glass jar (25 × 12 × 25 cm^3^) filled with 15 cm water at 24 ± 1°C No pretest	FST, TST EZM^q^, MCT^r^, MWMT, PAT^s^

a *Streptozotocin*.

b *Lipopolysaccharide*.

c *Identified as an outlier and removed from the meta analysis*.

d *Week of age at the beginning of the trial*.

e *Day of age at the beginning of the trial*.

f *Reversal: The Lactobacillus-based supplementation was applied after the stress treatment*.

g *Protection: The Lactobacillus-based supplementation was during before the stress treatment*.

h *Prevention: The Lactobacillus-based supplementation was applied before the stress treatment*.

i *Ø represent the diameter*.

j *Elevated Plus Maze*.

k *Open Field Test*.

l *Sucrose Preference Test*.

m *Novel Object Recognition*.

n *Light Dark Transition Task*.

o *Tail Suspension Test*.

p *Morris Water Maze Test*.

q *Elevate Zero Maze*.

r *Mirror Chamber Test*.

s *Passive avoidance Test*.

### Data Collection

Data relating to the effect of *Lactobacillus*-based supplementation and stress treatment on the time spent immobile in the FST were extracted using a custom-tailored form (excel spreadsheet). The form included information on study populations (sex, age, breed, and body weight), trial designs, *Lactobacillus* supplementations (*Lactobacillus* species and strains, dosage, and duration), stress treatments (category of stressor, frequency, and duration) and behavioral outcomes (group sample sizes, mean value of effect and group variance). The durations of the *Lactobacillus* supplementation and the stress treatments were categorized as acute ( ≤ 7 days) or chronic (>7 days).

When results were available only in graphical format, data were extracted using GetData Graph Digitizer software (GetData Graph Digitizer., GetData Graph Digitizer.). Graph digitization has been previously shown to be a valid method for extracting study data (Guyot et al., 2012). Time spent immobile in the FST was reported either in seconds (s) or as a percentage of the total test time, with the variance expressed in standard error or standard deviation. To homogenize the dataset, all data were transformed into seconds and the uncertainty was expressed as standard error. If the data were unclear or some key data were missing, we attempted to contact the corresponding authors through email to obtain further information. All steps (manuscripts screening and data extraction) were performed by a single reviewer.

### Statistical Model Development

All statistical analyses were completed using SAS® Studio University Edition. Preliminary calculations were completed in Microsoft® Excel® 2016. Statistical significance was considered at *P* < 0.05. Values are presented as least squares means (LSM) ± standard error (SE), unless stated otherwise. A mixed model approach was utilized for this meta-analysis (generalized linear mixed model) in order to adequately model the random effect of study (St-Pierre, 2001).

The outcome of this study is the FST immobility time in seconds. Initially, we assessed the effect of *Lactobacillus*-based supplementation (Model 1: Supplemented, Control) and stress (Model 2: Stress, Non-stress) on the outcome separately. Following this, the main and final model was built including the effects of *Lactobacillus*-based supplementation, stress and their interaction (Model 3). Generalized linear mixed models (proc glimmix) were used with the study included as a random effect. The following additional variables were added to the main model (Model 3) as covariates to assess their impact on the outcome: the study design (number of additional behavioral tests used, presence of a pretest, container's diameter and depth, water depth and temperature), the population (species, breed, sex and age), differences in the *Lactobacillus*-based supplementation treatment (number of strains used, concentration, duration, frequency and method of the supplementation), and differences in the stress treatment (category, duration, frequency) and whether the stressor was applied before (prevention), during (protection) or after (reversal) the supplementation (Table 1).

### Statistical Model Evaluation

The requirement for normally distributed and homogeneous studentized conditional residuals/random effect of study were examined graphically with the use of residual, histogram, and QQ plots. The covariance structure was chosen according to the goodness-of-fit statistics [Akaike information criterion (AIC) and Bayesian information criterion (BIC)]. Differences between LSMs were compared pairwise using a Tukey-Kramer adjustment for multiple comparisons. To detect possible study outliers, the Cook's distance was used with a 4/n cut-off, where n is the number of studies (Kutner et al., 2005). The mean difference (MD, MD_stress_= Mean_stress_ – Mean_non−stress_; MD_supplementation_= Mean_*Lactobacillus*_ - Mean_control_) and standard error of the difference (SED) were calculated to assess biases via funnel plots (Higgins and Thomas, 2019) and response heterogeneity using forest plots. When two or more *Lactobacillus*–based supplementation treatment groups were involved in one study (for example, with differing strain or dose), the data of the different groups were averaged as one group for funnel/forest plot analysis. Since the outcomes of recruited studies were continuous data, the 95% confidence intervals (CI) were calculated for statistical analyses and plotting. In the funnel/forest plots representing the effect of the stress and *Lactobacillus*-based supplementation treatment, a MD > 0 indicates that the stress treatment or *Lactobacillus*-based supplementation increased the FST immobility time. On the contrary, a MD <0 indicated that the stress treatment and the *Lactobacillus*-based supplementation decreased the length of immobility in the FST.

To evaluate the model's goodness-of-fit, scatter plots of residuals vs. adjusted (for the random effect of study) predicted values and predicted vs. observed values were plotted. The significance of the slope and intercept of the residual vs. adjusted predicted plots and interfering factors (covariates), were tested against zero and the slope/intercept of the predicted vs. observed plots against 1/0. These were evaluated in proc reg (SAS).

The impact of the *Lactobacillus*-based supplementation and stress treatment on the FST immobility time prediction equation(s) were evaluated using two methods.

Firstly, the mean square prediction error (MSPE) was calculated as

(1) $$\begin{array}{l} MPSE=\overset{n}{\underset{i=1}{\sum }}{\left(Oi-Pi\right)}^{2}/n \end{array}$$

where *n* is the total number of observations, *O*_*i*_ is the observed value, and *P*_*i*_ is the predicted value. The root mean square prediction error (rMSPE) was expressed as a percentage of the observed mean to give an estimate of the overall prediction error.

The rMSPE was then further decomposed into error due to overall bias (ECT), error due to deviation of the regression slope from unity (ER) and error due to the disturbance (random error) (ED) (Bibby and Toutenburg, 1977). The ECT, ER and ED fractions of MSPE were calculated as:

(2) $$\begin{array}{l} ECT={\left(P-O\right)}^{2}, \end{array}$$

(3) $$\begin{array}{l} ER={\left(S_{p}-R^{*}S_{o}\right)}^{2}, \end{array}$$

(4) $$\begin{array}{l} ED={\left(1-R^{2}\right)}^{*}S_{o}^{2},\; \end{array}$$

where *P* and *O* are the predicted and observed means, respectively, *Sp* is the predicted standard deviation, *S*_*o*_ is the observed standard deviation and *R* is the Pearson correlation coefficient. The ECT, ER and ED were then expressed as a percentage of the MPSE and sum to account for 100% of the error.

Secondly, concordance correlation coefficient analysis (CCC) was performed (Lin, 1989), where CCC was calculated as:

(5) $$\begin{array}{l} CCC=R^{*}C_{b}, \end{array}$$

where *R* is the Pearson correlation coefficient and *C*_*b*_ is a bias correction factor, the latter was calculated as:

(6) $$\begin{array}{l} Cb=\frac{2}{\left(V+\frac{1}{V}+u^{2}\right)},\; \end{array}$$

where

(7) $$\begin{array}{l} V=\frac{So}{Sp},\; \end{array}$$

and

(8) $$\begin{array}{l} \text{u}=\frac{O-P}{{\left(So^{*}Sp\right)}^{1/2}},\; \end{array}$$

## Results

### Preliminary Assessment of the Data

**Figure 2:**
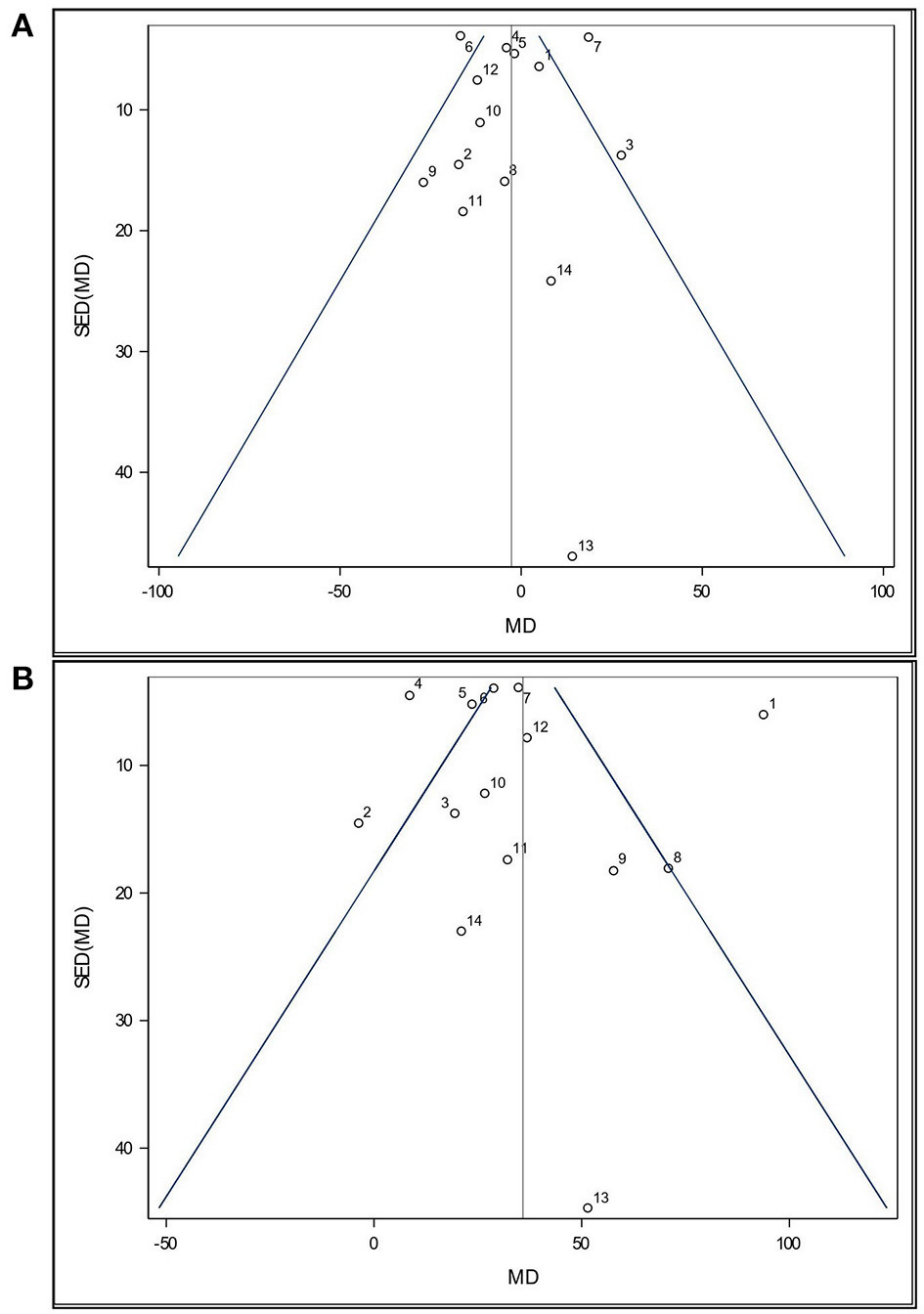
Funnel plot of the effects of **(A)** *Lactobacillus*-based probiotic supplementation and **(B)** stress on time spent immobile (s) in the Forced Swim Test in rodents for all included studies (*n* = 14). Dots represent mean difference (MD, *Lactobacillus*-based probiotic—Control; stress—non stress) and standard error of the mean difference [SE(MD)] for each study, blue lines represent 95% CI, and the black line represents the overall fixed effect average.

Funnel plots (Figures 2A,B) were used to visually assess the dataset for publication bias. Such plots presume that data should exist within a 95% confidence funnel, whereby highly precise studies with a low standard error of the difference (SED) should exist close to the average/true effect size (vertical line) and that as SED increases so will the scatter of those points around the average/true effect size. Each point within a funnel plot represents a study's mean difference (MD).

Both funnel plots are partially symmetrical, indicating no obvious evidence of asymmetry, and, therefore, no evidence of publication bias. Both funnel plots show that all studies were comparatively precise. One study (Wei et al., 2019, Study 13) was found to have a higher SED relative to all others in this analysis. This was due to a much higher SE in all their treatments groups. While study 13 appeared less precise, the MD value is still close to the overall effect size (Figures 2A,B). Since this study was not reported as an outlier by Cook's distance, it was retained in the analysis. Additionally, three studies showed a small SE compared to their large stress effect size (Study 1, 2, and 4). However, no distinction in experimental design was found.

**Figure 3:**
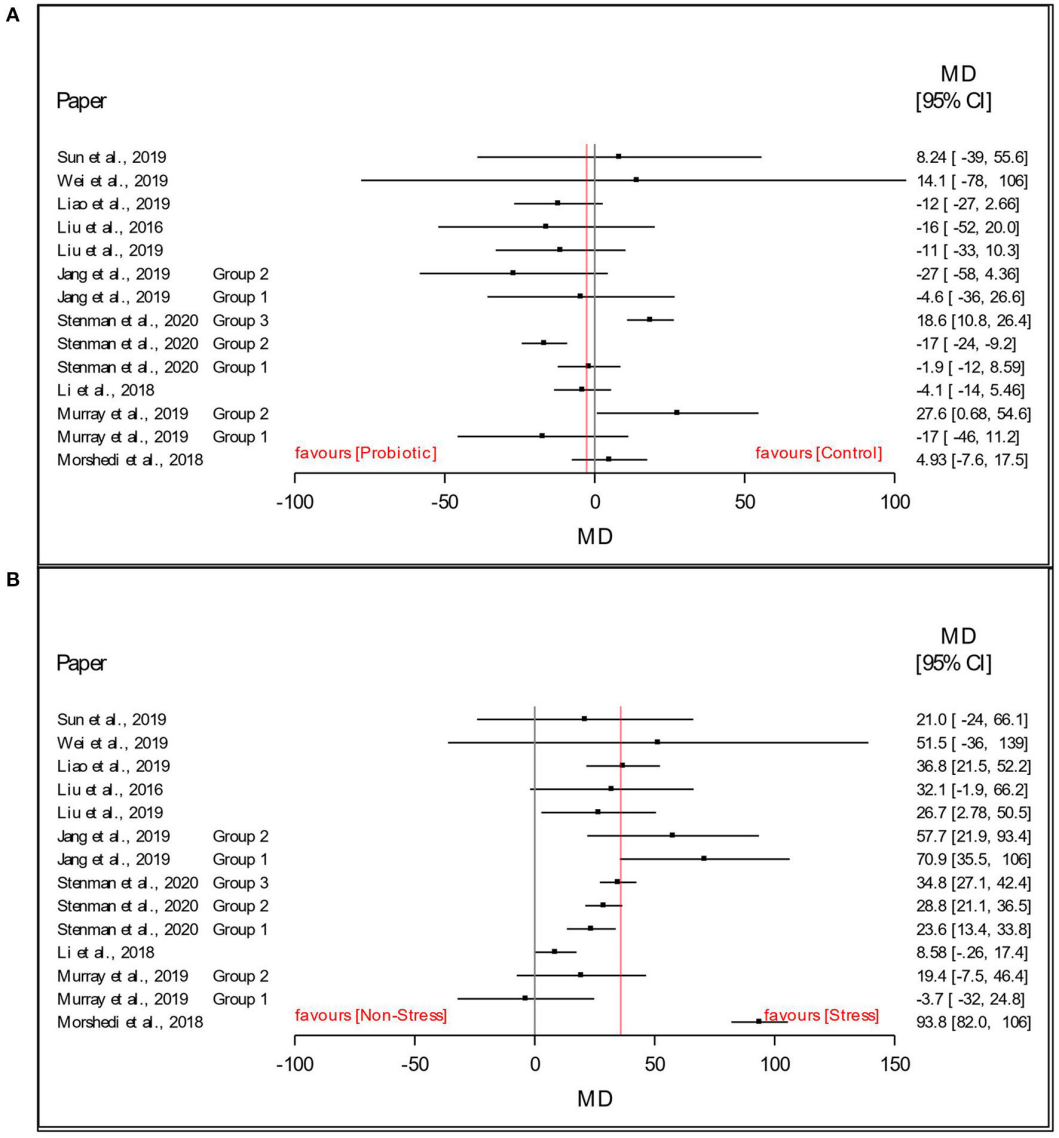
Data and forest plot of the effect of **(A)** *Lactobacillus*-based probiotic supplementation and **(B)** stress on time spent immobile (s) in the Forced Swim Test in rodents for all included studies (*n* = 14). Dots represent mean difference (MD, *Lactobacillus*-based probiotic—Control; stress—non stress) for each study, lines represent 95% CI, and the red line represents the overall fixed effect average.

As a preliminary analysis and visualization of the dataset, Figures 3A,B present a forest plot of the stress and probiotic effect sizes (mean difference, MD: stress - non-stress; *Lactobacillus*-based supplementation - Control) by study with 95% confidence intervals. In Figure 3A, this preliminary visual assessment shows that the average MD_stress_ of the FST immobility time was positive, indicating that stressed animals had a higher average immobility time in the FST. In Figure 3B, visual assessment shows that the average MD_supplementation_ of the immobility time in the FST was slightly below 0, indicating that the animals receiving the *Lactobacillus*-based supplementation treatment decreased the time spent immobile in the FST compared to those receiving the control supplement. Such plots and averages represent the fixed effect analysis of Model 1 and Model 2 and a high-level visual examination of the data.

### Meta-Analysis Model Predictions

The results of the three models developed (supplementation alone, stress alone, supplementation × stress) across studies are presented in Table 2. Model 1 shows no effect of the *Lactobacillus*-based supplementation on time spent immobile in the FST [Model 1, (95% CI) −9.75 to 15.52, F_1,35_ = 0.21; *P* = 0.647]. Model 2 shows a significant effect of the stress treatment [Model 2, (95% CI) −33.68 to −11.09, F_1,37_ = 16.13; *P* < 0.001], whereby stress treatment significantly increased the duration of immobility in rodents within the FST. Model 3 shows a significant interaction of supplementation and stress treatments (Model 3, F_1,35_ = 14.68; *P* < 0.001). Supplementation with *Lactobacillus*-based probiotics mitigated the immobility duration in stressed rodents [Supplemented-Stress: 130.0 ± 17.82 s vs. Control-Stress: 152.7 ± 17.94 s, (95% CI) 12.23–33.07, *P* < 0.001]. However, *Lactobacillus*-based probiotics did not significantly impact immobility duration in the non-stressed populations [Supplemented-Non-Stress: 135.9 ± 19.89 s vs. Control-Non-Stress: 114.30 ± 17.94 s, (95% CI) −42.33 to −0.87, *P* = 0.168]. Based on the above results, we conclude that across 14 studies, *Lactobacillus*-based supplementation alone did not impact the duration of immobility in the FST (Model 1) while a stress treatment alone increased the duration of immobility (Model 2). Our main model (Model 3) showed similar results but, more importantly, it highlighted that the *Lactobacillus*-based supplementation reduced the duration of immobility in the FST in the stressed rodent population compared to the control stressed population. The *Lactobacillus*-based supplement had no effect on the non-stressed populations.

**Table 2:** Predictive equation of the immobility duration [in seconds (Y)] spend in the Forced Swim Test (FST) based on the *Lactobacillus*-based supplementation and stress treatments in rodents.

**Model**	**Fixed effect**	**Equation: estimate (s) ± standard error**	***P*-value**
Model 1: Supplementation	Control (*n* = 28)	Y = 133.47 ± 17.7913 a	0.6469
	Supplemented (*n* = 24)	Y = 130.59 ± 17.9145 a	
Model 2: Stress	Non-Stress (*n* = 17)	Y = 117.28 ± 18.0394 a	0.0003
	Stress (*n* = 35)	Y = 139.66 ± 17.7479 b	
Model 3: Supplementation * Stress	Control Non-Stress (*n* = 14)	Y = 114.30 ± 17.9364 b	0.0005
	Control Stress (*n* = 14)	Y = 152.65 ± 17.9364 a	
	Supplemented Non-Stress (*n* = 3)	Y = 135.90 ± 19.8877 ab	
	Supplemented Stress (*n* = 21)	Y = 130.00 ± 17.8201 b	

*Different letters indicate statistically significant differences within the same Model (P < 0.05)*.

### Potential Impact of Covariates on the Duration of Immobility

**Figure 4:**
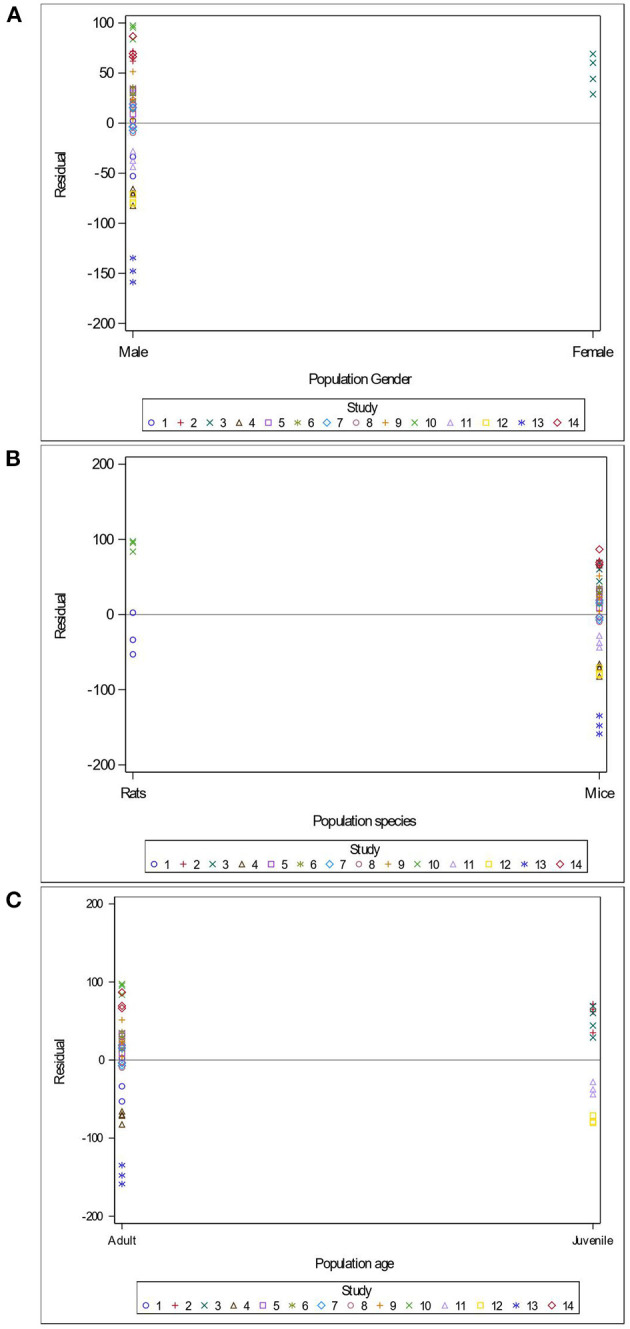
Raw residuals of Model 3 (Supplementation*Stress) plotted against the sex **(A)**, species **(B)** and age **(C)** of the population, where points represent treatment means and are coded by study.

**Figure 5:**
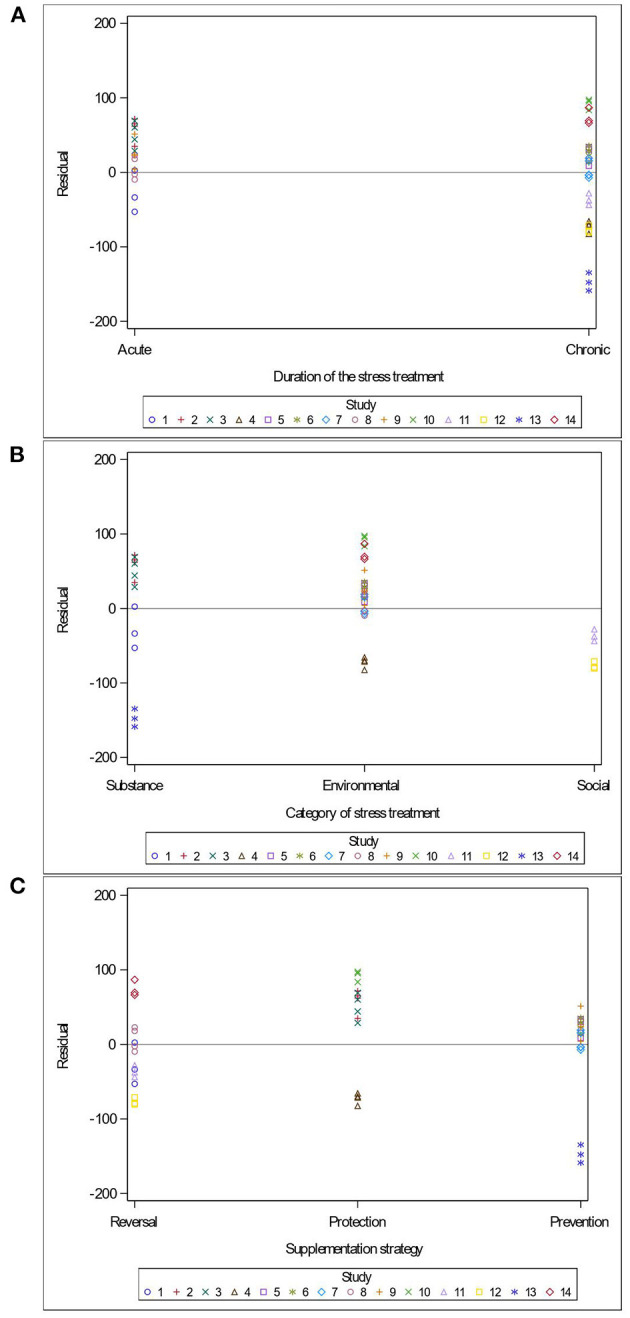
Raw residuals of Model 3 (Supplementation*Stress) plotted against duration of the stress treatment **(A)**, category of stressors **(B)**, the treatments order **(C)** where points represent treatment means and are coded by study.

**Figure 6:**
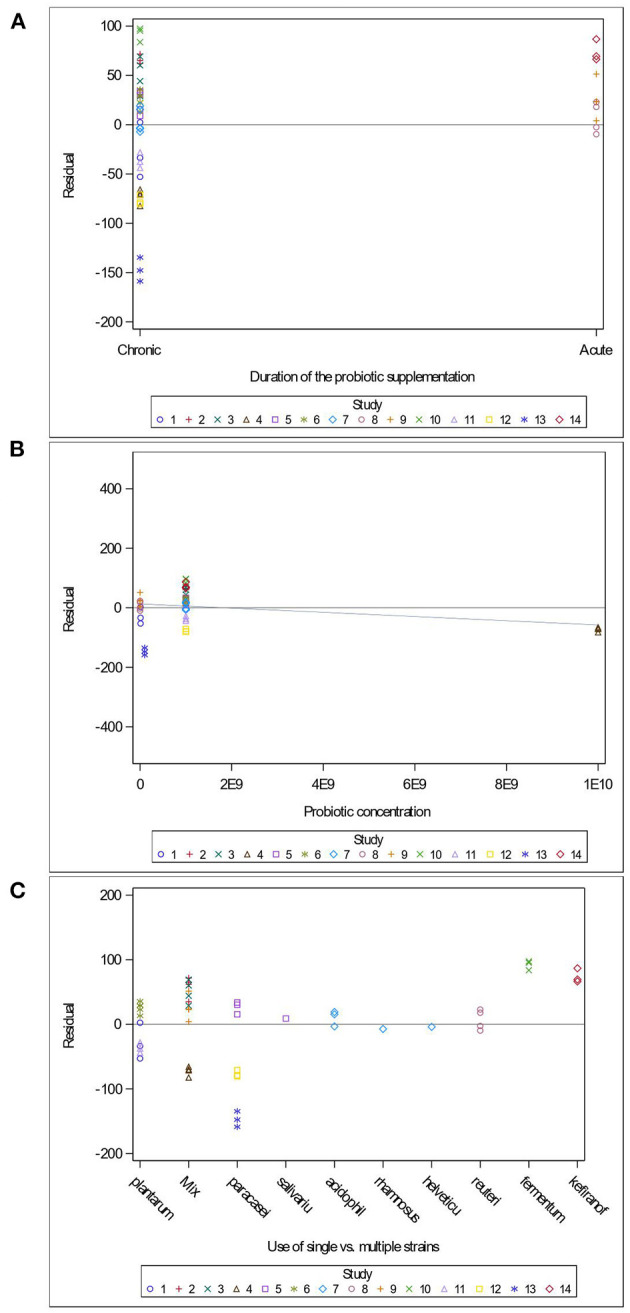
Raw residuals of Model 3 (Supplementation*Stress) plotted against the duration of *Lactobacillus*-based probiotic supplementations **(A)**, probiotic concentration **(B)** and the use of single or combination of bacterial strains as probiotics **(C)**, where points represent treatment means and are coded by study.

Variables related to the study design (presence of a pretest, container's diameter and depth, water depth and temperature), the rodent population (species, breed, sex and age), the stress treatment (duration, frequency, category, order of administration relative to the supplement), and the *Lactobacillus*-based supplementation (specific bacterial strain, supplement duration and bacterial dosage) were tested as potential sources of variation in predicting immobility time in the FST. The tested variables were not significant when added to Model 3 as covariates (*P* > 0.05). These variables were not kept in Model 3, but to further illustrate their impact, the (marginal) residuals of Model 3 were plotted against these covariates (population species, sex and age; stress treatment duration, category and order; *Lactobacillus*-based supplementation duration, dosage and strains) (Figures 4–6).

There is a clear and unequal distribution of sex (Figure 4A) and species (Figure 4B) of the rodents across our data. Indeed, only one study in our meta-analysis utilized females (Murray et al., 2019) and only two studies assessed rats (Morshedi et al., 2018; Liu et al., 2019). The female study showed a tendency to over-predict the immobility duration in the FST (Figure 4A). The residuals were balanced in studies using rats and mice (Figure 4B). In contrast, data were distributed equally across age (adult vs. juvenile), and residuals in adults (10 studies) and in juveniles (four studies) appear balanced (Figure 4C).

The duration of the stress treatment showed balanced residuals across both acute and chronic stressors (Figure 5A). Three main categories of stress (injection of a substance, environmental stress and social stress) were used in the 14 studies of this meta-analysis. While predictions are balanced for the substance injections and environmental stress, the two studies using maternal separation as a social stressor under-predicted immobility duration (Figure 5B). Residuals were balanced regardless of the order in which studies supplemented or stressed their population (Figure 5C).

The duration of the *Lactobacillus*-based supplementation is also unequally distributed across our data. Indeed, only three studies (Jang et al., 2019; Sun et al., 2019) administered supplements short-term, which resulted in over-predicting the immobility duration in the FST (Figure 6A). The probiotic dose within the studies used in the meta-analysis ranged between 1 × 10^7^ and 2 × 10^10^ colony forming units (CFU) (Figure 6B). Only one study (Dhaliwal et al., 2018) supplied a 2 × 10^10^ CFU dose, which under predicted the duration of immobility in the FST (Figure 6B). Our analysis consisted of seven studies using a single bacterial strain, while the remaining seven used a combination of two to four strains, and no more than three studies used the same strain. However, the predictions for the studies using the same strains (*L. paracasei* and *L. plantarum*) and studies using combinations of strains are balanced (Figure 6C).

The number of other additional behavioral tests (e.g., sucrose preference test or tail suspension test, Table 1) used for each of the 14 studies was also tested as a covariate in the Model 3 and was found to significantly impact the immobility duration in the FST (*P* = 0.0188) without changing the overall impact of the supplementation or stress. To further interpret this impact, we incremented Model 3 separately (deemed Model 4; not reported) to assess the interaction between the number of other behavioral tests and the *Lactobacillus*-based supplement, as well as the interaction between the number of other behavioral tests and the stress treatment on the outcome.

We report that, while the interaction between the number of other behavioral tests and the *Lactobacillus*-based supplementation was not significant (*P* = 0.2469), the interaction between the number of other additional behavioral tests and the stress treatment was significant in determining the immobility duration in the FST (*P* = 0.0309). The addition of this variable to the model did not change the overall impact of the supplementation or stress treatment compared to Model 3. Furthermore, there was a low number of observations (*n* = 1) in the treatment groups, and the order in which the other tests were administered were not always specified. Thus, the variable was not retained, and Model 3 was selected as the final model.

### Model Evaluation

**Figure 7:**
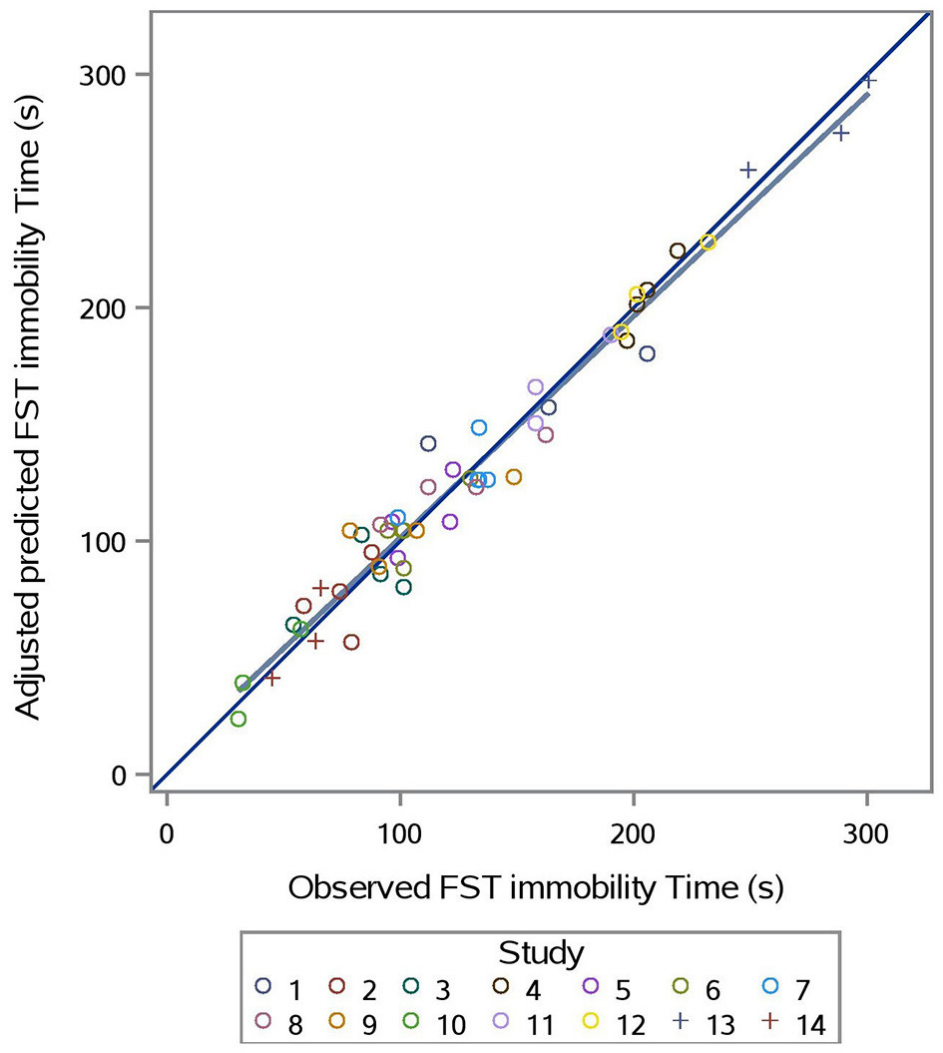
Adjusted predicted vs. observed FST immobility time (s) for prediction equation used in Model 3 (Supplementation*Stress).

**Figure 8:**
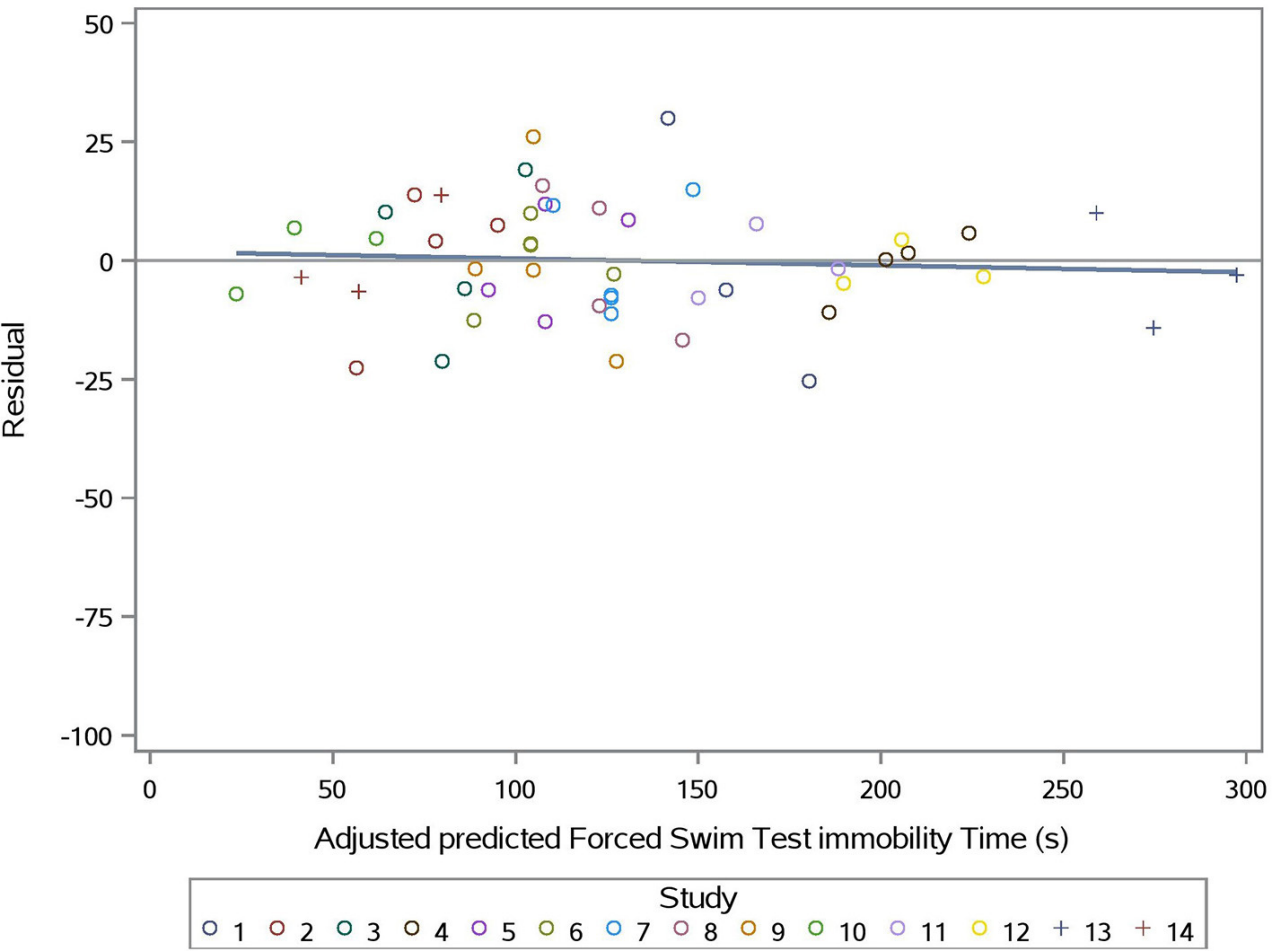
Adjusted predicted FST immobility time (s) vs. Residual for prediction equation used in Model 3 (Supplementation*Stress).

The predicted vs. observed and predicted vs. residual plots of Model 3 are presented in Figures 7, 8, respectively. The predicted values are conditional and adjusted for the random effect of study. Results show good agreement between predictions and observations, indicating that the model described the between- and within- study variation well. Assessment of the residual vs. adjusted predicted values showed no slope or mean bias (*P* > 0.05) (Figure 7).

Evaluation of Model 3 predictions via rMSPE and CCC analysis (Table 3) shows good statistical agreement between predictions and observations, which were typically both very precise (R) and accurate (C_b_), with a CCC value of 0.981 and an rMSPE value of 9.293% (Table 3).

**Table 3:** Results of root mean square prediction error (rMSPE) and concordance correlation coefficient (CCC) analysis of the Model 3.

**Model evaluation parameters**	**Model 3**
rMSPE %^a^	9.293
ECT %^b^	0.000
ER %^c^	0.528
ED %^d^	99.47
CCC^e^	0.981
R^f^	0.981
C_b_^g^	0.999
V^h^	1.034
μ^i^	0.000

a *Root mean square prediction error, as a percentage of observed mean*.

b *Error due to mean bias, as a percentage of total MSPE*.

c *Error due to regression, as a percentage of total MSPE*.

d *Error due to disturbance, as a percentage of total MSPE*.

e *Concordance correlation coefficient, where CCC = r × C_b_*.

f *Pearson correlation coefficient*.

g *Bias correction factor*.

h *Scale shift*.

i *Location shift*.

## Discussion

This study systematically reviewed and performed a meta-analysis of published randomized control trials (RCTs) assessing the effect of *Lactobacillus*-based probiotics and stress treatments on the duration of immobility in the Forced Swim Test (FST) in rodents. We conclude that *Lactobacillus*-based probiotics significantly reduced immobility in stressed rodents by 15% relative to a control treatment in the context of the FST. Previously published studies have suggested that ingesting specific strains of *Lactobacillus* species decreases anxiety- and depression-related behavior (Bravo et al., 2011; Mackos et al., 2013; Ohland et al., 2013) and provides a protective effect during stress (Gareau et al., 2007; Mackos et al., 2013, 2016). Our findings are also consistent with previous meta-analyses, which demonstrate compelling, alleviating effects of probiotics on human psychological disorders (Huang et al., 2016; McKean et al., 2017; Wallace and Milev, 2017). Consequently, it is tempting to postulate that bacteria belonging to the *Lactobacillus* genus may help to alter some behavioral traits manifested in psychological disorders.

Depression is a multifactorial disorder in humans. Its core symptoms include a depressed mood, a feeling of worthlessness, and recurring thoughts of death or suicide which are impossible to model in laboratory animals. The original version of the FST was designed to be “a primary screening test for antidepressants” (Porsolt et al., 1977). A drug's ability to reduce passive coping (i.e., decrease the duration of immobility) in the FST was found to be a predictor of its efficacy as an antidepressant in humans (Petit-Demouliere et al., 2005; Castagné et al., 2011). However, the FST's successful predictive validity for antidepressants has led to the anthropomorphic over-interpretation of its outcomes, whereby increased immobility is equated to behavioral despair or “depressive-like” traits (Molendijk and de Kloet, 2015; Yankelevitch-Yahav et al., 2015; De Kloet and Molendijk, 2016).

Therefore, while our results suggest that *Lactobacillus* bacteria may be effective in alleviating some depression symptoms, it should be noted that the FST is not analogous to the disorder in its entirety (Commons et al., 2017). Indeed, each standardized test (e.g., FST, tail suspension tests, sucrose preference) aimed at measuring various aspects of depression has its own strengths and weaknesses in terms of predictive, face and construct validities (Powell et al., 2012). The FST is one of the most commonly used test and a valid tool to assess antidepressant effects (Kara et al., 2018) due to its low costs and high reliability (Petit-Demouliere et al., 2005). Additionally, the FST has good predictive validity, and the analogy between the FST model and the disorder affords some face validity (Willner, 1984). Nevertheless, it holds poor construct validity and lacks specificity which can be explained by high sensitivity to methodological variations (Petit-Demouliere et al., 2005). Indeed, the validity of the FST when comparing doses and various compounds might only be relevant to single experiments (Kara et al., 2018). Furthermore, FST responses can also vary greatly depending on breed of animals (Petit-Demouliere et al., 2005; Powell et al., 2012). Considering the limits of this behavioral test, the suggestion that *Lactobacillus* may reduce depressive-like behavior under stress conditions should be interpreted with caution. In general, future research should focus on assessing a broader category of depression-associated behaviors and physiological parameters to elucidate the true impact of probiotic therapies and their efficiency in alleviating symptoms of psychological disorders. Therefore, further, rigorous investigation in humans and animal models that better represent the physiological and behavioral hallmarks of clinical depression is needed.

Keeping the above limitations regarding FST in mind, it is nevertheless noteworthy that the current literature on gut microbiome research provides several modes of action through which effective probiotic bacteria may exert effects on psychological disorders. A complex network, including the enteric nervous system (ENS), sympathetic and parasympathetic branches of the autonomic nervous system, neuroendocrine signaling pathways, and the neuro-immune system, supports communication between the gut and the central nervous system (CNS) (Grenham et al., 2011). The gut microbiota can, therefore, influence the CNS via the immune system and ENS, especially under conditions of stress (Foster and McVey Neufeld, 2013; Huang et al., 2016; Foster et al., 2017; Wallace and Milev, 2017). Indeed, stress increases intestinal permeability (Maes et al., 2009) allowing commensal microorganisms to translocate across the intestinal mucosa and interact with both immune cells and ENS neurons. This “leaky gut” effect has been associated with major depressive disorder (MDD) (Gareau et al., 2008; Teitelbaum et al., 2008). More specifically, the increase of the pro-inflammatory mediators IL-6 and IFN-γ in response to bacterial translocation across the gut epithelium (Foster et al., 2017) is also mirrored in depressed patients (Hodes et al., 2015). Importantly, *Lactobacillus* supplementation is demonstrated to suppress the pro-inflammatory response and protects against the disruption of the gut barrier by producing compounds that promote its integrity (Matsumoto et al., 2005; Lin et al., 2008; Ait-Belgnaoui et al., 2012; Kozakova et al., 2016). This group of bacteria has also been implicated in GABA receptor expression via the vagus nerve (Bravo et al., 2011), hyperpolarization of ENS neurons, influencing gut motility and pain perception via the ENS (Kunze et al., 2009), and increasing dopamine and serotonin levels (Liu et al., 2016). It is conceivable that these connections between the gut microbiota and the CNS may be disrupted in depression. Given the link between this bacterial genus and key CNS compounds, *Lactobacillus* supplements could be used as a tool to increase our knowledge and understanding of the interactions within the complex gut-brain network, and to, eventually, modulate its function. This putative connection to psychological disorders may also explain why *Lactobacillus*-based supplementation was most effective at reducing immobility time in the FST in rodents that were more stressed. It is likely that the benefits imparted to rodents that did not undergo an additional experimental stressor are limited.

Remarkably, the time and the order in which studies supplemented or stressed their population did not impact the outcome of the FST (Figure 5C). This is in agreement with Hadizadeh et al. (2019), who demonstrated that offspring of rat dams stressed during their third trimester showed marked improvements in mood disorders symptoms when supplemented with a *Lactobacillus*-based probiotic either as a prevention (pre-stress) or as a reversal (post-stress) treatment. Taken together with our findings, the data suggests that probiotic supplementation can be both preventive (when supplemented before a stress) and protective (when supplemented during a stress). It further suggests that *Lactobacillus*-based probiotic can reverse pathophysiological changes associated with some psychological disorders.

In addition, we further report that acute (i.e., short term) *Lactobacillus* supplementation led to over-predicting the duration of immobility in the FST (Figure 6A). While the ideal treatment duration to observe a positive impact of a probiotic is unknown (Wallace and Milev, 2017), the adherence of bacteria to epithelial cells and mucosal surfaces is a crucial criterion for probiotic selection (Duary et al., 2011). Goldin et al. (1992) reported that a *Lactobacillus* strain persisted in human feces for up to 4 days in 87% of individuals, but up to 7 days in 33% of the individuals. This suggests that the gut transit and retention times may ultimately influence treatment efficacy, and that individual differences between subjects can influence probiotic gut colonization. Further investigation is necessary to better assess the impact of single strain supplementation, as well as the synergistic impact of strains on stress-induced behaviors.

It is noteworthy that the non-stressed individuals of each study were not completely devoid of stress. Indeed, five studies conducted the FST based on the original protocol from Porsolt et al. (1977), which implement a pretest session 24 h prior to data collection (48 h in 1 study, Wei et al., 2019, Study 13). This preconditioning step represents an additional stressor thought to induce a state of behavioral despair (Porsolt et al., 1977). Indeed, the pretest modifies the behavior of animals as rats develop immobility significantly faster during the second test (Porsolt et al., 1977). Thus, comparing studies that used a pretest to those that did not may yield different results. In order to account for this, the impact of the presence or absence of a pretest was tested in this meta-analysis as a covariate. However, we report no impact on the immobility outcome in the FST (*P* > 0.05). Additionally, all 14 studies included in this meta-analysis assessed animals' behavior and cognition through test batteries (between 2 and 5 tests, including the FST, Table 1). The actual order in which the tests were carried was not always specified, which implies that numerous handling procedures and stressful situations for the animals may have taken place prior to the FST. While short-term, daily handling for 5 days prior to the FST may not affect immobility time in rats (Bogdanova et al., 2013), research shows that the choice of handling method (e.g., picking up by the tail, use of tunnels or open hand voluntary approach) can induce profound variations in the mice's stress response (Hurst and West, 2010). Indeed, gentle handling reduced immobility duration in the FST compared to mice that were aversively or minimally handled (Neely et al., 2018). Unfortunately, the techniques or quality of handling is sparsely reported in publications, even in behavioral studies.

We also tested the impact of other behavioral testing, in addition to the FST, on the outcome. The number of other behavioral test appeared to significantly influence the immobility duration (data not shown). However, this consideration should be taken with caution as there was a low number of observations (*n* = 1) in the treatment groups, and the order in which the other tests were administered were not always specified. Thus, it is unknown whether these other tests happened before or after the FST, which is crucial for the interpretation of the results. We assume that only behavioral tests and the amount of handling happening before the FST would impact the result in the FST. We cannot exclude the possibility that the additional handling and behavioral testing represented yet another stressor, and it is possible that these test batteries may have masked the true changes in immobility duration in response to the intended stress treatment. Thus, all individuals in the studies would have been subjected to accumulated stress over the course of the experiments and various testing procedures, regardless of the intended stress regimen. As such, a limitation of this meta-analysis is that the non-stressed groups are not fully devoid of a stressful environment prior to the FST. Nevertheless, given that all animals in a given study underwent the same behavioral tests, the observed differences between the groups would still remain indicative of the impact of the intended stress regimen on the FST outcomes (Table 1).

Surprisingly, we report that key variables such as the age, sex, animal species and breed, *Lactobacillus* strain and bacterial dosage did not significantly affect the FST immobility duration in our main model. It is noteworthy that male animals are preferentially selected for animal models due to concerns about confounding contributions from the oestrous cycle, which is known to influence behavior in the FST (Bogdanova et al., 2013). The only study within the meta-analysis that employed both male and female subjects did not control for the ovarian cycle phase (Murray et al., 2019). Mice received the *Lactobacillus*-based probiotic treatment during their pubertal period of development (5–7 weeks of age), which may have impacted the results of that study. We report a significant sex-dependent over-prediction of the duration of immobility based on the residuals for female subjects, an observation which is absent in males (Figure 4A). This suggests that the female animals used in Murray et al. (2019) were tested during the proestrous or estrous phases as longer immobility times in the FST are observed within these periods (Bogdanova et al., 2013). This sex difference is in line with the current literature on affective disorders, where rates of depression are distinctly higher in females (Cáceda et al., 2014; Parker et al., 2014; World Health Organization, 2017). Indeed, women have higher rates of depression and often experience depression symptoms during critical reproductive periods (Noble, 2005). These sex-based significant differences highlight the need for rigorously controlled studies, including a balance between both sexes and a control of the female oestral cycle phase, to improve sex-oriented treatment of depression (Clayton and Collins, 2014; Tannenbaum et al., 2016). Similar to the unbalanced use of males and females across studies, we report that only two studies used rats (Morshedi et al., 2018; Liu et al., 2019), while the remaining studies used mice. Rodents are some of the most-widely used animal models, but they present several species-specific similarities and differences within the gut microbiota (Nagpal et al., 2018). Interestingly, mice microbiota are more similar to humans than those in rats (Nagpal et al., 2018). Because of the variability of these species-specific gut microbiota signatures, it is unclear whether the same *Lactobacillus*-based supplementation would impact each rodent species the same way. Our meta-analysis shows that mice and rats display similar immobility behavior in the FST. Indeed, Figure 4B shows balanced residuals in both rats and mice. The number of publications assessing the influence of the same *Lactobacillus*-based supplementation in multiple species is limited. Yet, in a double-blind RCT, daily supplementation with a combination of *Lactobacillus helveticus* R0052 and *Bifidobacterium longum* displayed beneficial psychological effects both in rats and in humans (Messaoudi et al., 2011). Thus, some *Lactobacillus*-based supplementations are beneficial across species, despite the innate gut microbiota disparities. This hypothesis should be further tested to increase our understanding.

Behavioral responses to FST are also proposed to depend on the water temperature (Bruner and Vargas, 1994; Bogdanova et al., 2013). Indeed, rodents swimming in relatively cold water (usually 25°C) compared to their core body temperature (~36–37°C) show increased motor activity, as well as changes in cardiovascular and neurochemistry parameters (Porsolt et al., 1979; Linthorst et al., 2008). However, the water temperature did not impact the immobility time in the FST (*P* > 0.05) within this meta-analysis as the studies used similar water temperatures (22–25°C, Table 1). Similarly, the container dimensions of the FST set-up play a significant role in determining immobility behavior. When first developed, the FST tank was designed to have a depth of 15 cm of water, shallow enough for the rat to feel the bottom with its hind paws and tail (Porsolt et al., 1978). Numerous modified versions of the FST involve containers of various diameters and water depth which may alter swimming and immobility behavior. In the present meta-analysis, the water depth (varying between 10 and 30 cm), and the container dimensions (diameter varying between 9 and 21 cm and depth between 25 and 50 cm) did not impact the immobility time in the FST (*P* > 0.05).

To the best of the authors' knowledge, this study is the first to systematically review the effect of *Lactobacillus*-based probiotics on the duration of immobility in an FST in rodents by pooling the results of RCTs. Nevertheless, it is important to note a few limitations of this work. Indeed, while some population parameters, the probiotic strain, the dose and the type of stress treatment were recorded and considered in this analysis, other factors that were not considered, such as diet, may also significantly contribute to immobility in the FST. In particular, there is great individual variability between healthy gut microbiomes, which may influence responses to supplementation with particular strains of probiotics.

Additionally, the number of studies included in this analysis and the relative sample sizes of the studies are also somewhat limited. While the current analysis only showed a significant impact of *Lactobacillus*-based probiotics on the immobility behavior of stressed rodents, it is important to note that only two studies measured the outcomes in a non-stressed treatment group (Li et al., 2018; Murray et al., 2019), which may have influenced the results. Previously published meta-analyses have reported a positive effect of Lactobacilli on psychological symptoms of depression in healthy, non-depressed humans (Messaoudi et al., 2011; Huang et al., 2016; McKean et al., 2017) and highlights the need for more rigorous RCTs in non-stressed individuals. We also observed some large-scale differences of the mean differences between all studies (Figure 3). However, the visual assessment of funnel plots revealed no publication bias (Figures 2A,B). Finally, it should be acknowledged that the manuscript search, screening and data extracting were performed by a single reviewer and that the protocol has not been stored in a public repository. However, the steps in the review protocol are presented in the current study allowing for replication. The single reviewer received oversight from the co-authors, but it may nonetheless have impacted data quality and accuracy, including the omission of studies published in languages other than English.

## Conclusions

This systematic review and meta-analysis integrate results of previous studies and support the potential role of *Lactobacillus*-based probiotics in mitigating stress-induced behaviors. Within the context of the FST, we report that *Lactobacillus*-based probiotic supplements significantly decreased the duration of immobility of stressed rodents. Further evidence from larger samples and more rigorous randomized control trials are needed to further elucidate the influence of important biological factors such as sex, animal species, duration of supplementation, *Lactobacillus* species, and the order of supplementation and stress treatments. The impact of *Lactobacillus*-based probiotic supplements under non-stressful conditions should also be evaluated in depth. The potential use of probiotics as a novel treatment or prevention strategy for major psychological disorders may aid in avoiding the stigma, latency and side effects associated with current antidepressants usage.

## Data Availability Statement

The raw data supporting the conclusions of this article will be made available by the authors, without undue reservation.

## Author Contributions

CM, JE, NS, and AH-M: conceptualization, methodology, and writing—review and editing. CM and JE: formal analysis and investigation. CM: writing—original draft. JE, NS, and AH-M: supervision. AH-M: funding acquisition. All authors contributed to the article and approved the submitted version.

## Conflict of Interest

The authors declare that the research was conducted in the absence of any commercial or financial relationships that could be construed as a potential conflict of interest.
